# Role of Segregation for Variant Discovery in Multiplex Families Ascertained by Probands With Left Sided Cardiovascular Malformations

**DOI:** 10.3389/fgene.2018.00729

**Published:** 2019-01-11

**Authors:** Lisa J. Martin, Valentina Pilipenko, D. Woodrow Benson

**Affiliations:** ^1^Department of Pediatrics, Cincinnati Children’s Hospital Medical Center, Cincinnati, OH, United States; ^2^Department of Pediatrics, University of Cincinnati School of Medicine, Cincinnati, OH, United States; ^3^Department of Pediatrics, Medical College of Wisconsin, Milwaukee, WI, United States

**Keywords:** linkage, heart, complex trait, exome, gene

## Abstract

Cardiovascular malformations (CVM) are common birth defects (incidence of 2–5/100 live births). Although a genetic basis is established, in most cases the cause remains unknown. Analysis of whole exome sequencing (WES) in left sided CVM case and trio series has identified large numbers of potential variants but evidence of causality has remained elusive except in a small percentage of cases. We sought to determine whether variant segregation in families would aid in novel gene discovery. The objective was to compare conventional and co-segregation approaches for WES in multiplex families. WES was performed on 52 individuals from 4 multiplex families ascertained by probands with hypoplastic left heart syndrome (HLHS). We identified rare variants with informatics support (RVIS, minor allele frequency ≤0.01 and Combined Annotation Dependent Depletion score ≥20) in probands. Non-RVIS variants did not meet these criteria. Family specific two point logarithm of the odds (LOD) scores identified co-segregating variants (C-SV) using a dominant model and 80% penetrance. In families, 702 RVIS in 668 genes were identified, but only 1 RVIS was also a C-SV (LOD ≥ 1). On the other hand, there were 109 non-RVIS variants with LOD ≥ 1. Among 110 C-SV, 97% were common (MAF > 1%). These results suggest that conventional variant identification methods focused on RVIS, miss most C-SV. For diseases such as left sided CVM, which exhibit strong familial transmission, co-segregation can identify novel candidates.

## Introduction

Cardiovascular malformations (CVM) are the most common birth defects with an incidence of 2 to 5 per 100 live births (Benson, 2002). CVM occur during cardiogenesis, and phenotypes and clinical impact are varied. HLHS is a severe form of CVM characterized by hypoplasia of the left ventricle and ascending aorta in addition to atresia or hypoplasia of the aortic and mitral valves. While rare (prevalence 0.02%) (Parker et al., 2010), it accounts for 25% of infant deaths due to CVM (Boneva et al., 2001). On the other hand, bicuspid aortic valve (BAV) is the most common CVM, affecting 1–2% of the population. The prevailing view is that BAV and HLHS are developmentally related, and that the two phenotypes represent extremes of a spectrum of CVM involving structures on the left side of the heart; related phenotypes include abnormalities of the aorta, mitral valve and heart chamber septa (Brenner et al., 1989; Loffredo et al., 2004; McBride et al., 2005; Hoang et al., 2018). Although CVM heritability, especially for left sided CVM, provides strong evidence of a genetic basis (Cripe et al., 2004; McBride et al., 2005; Hinton et al., 2007; McBride and Garg, 2011; Hanchard et al., 2016; Kuo et al., 2017), in most cases the cause remains unknown. Exome data for left sided CVM case series and trios has identified large numbers of potential variants but definitive evidence of causality has remained elusive except in a small percentage of cases (Russell et al., 2018).

As originally described by Ng et al. (2010a; 2010b), the conventional approach to variant discovery for exome sequencing prioritizes variants which occur rarely in population databases and are predicted to impact protein structure. Current American College of Medical Genetics (ACMG) clinical guidelines (Richards et al., 2015) recognize these criteria but also provide additional criteria for pathogenicity including biologic rather than *in silico* support for variant functionality and a role of the gene in disease etiology. Characterizing biologic support can be time consuming, costly, and challenging (Hosseini et al., 2018). Even with this approach, 2–11% of unaffected individuals harbor pathogenic variants in clinically actionable genes (Dorschner et al., 2013; Tabor et al., 2014; Lindor et al., 2017).

Another strategy to identify variants relies on linkage analyses to quantify co-segregation evidence (Martin et al., 2014; Richards et al., 2015). While inheritance within a trio has been used to prioritize variants identified using the conventional approach, formal quantification of segregation evidence through linkage analyses is not typically performed with next generation sequence data. As linkage analyses in multiplex families was used to identify CVM genes including *TBX5* (Basson et al., 1995, 1997), *NKX2.5* (Schott et al., 1998), and *NOTCH1* (Garg et al., 2005), we hypothesized that evaluation of co-segregation of variants and disease in a kindred using linkage analysis will aid in identifying the genetic underpinnings of disease such as left sided CVM that exhibit family clustering.

## Materials and Methods

### Study Population

We selected 4 multiplex families (*n* = 52 participants; Table 1) recruited as part of a family based genetic study of hypoplastic left heart syndrome (HLHS) (Hinton et al., 2007, 2009). Families were ascertained by a proband with HLHS. Additional, family members were recruited using a sequential sampling strategy. Briefly, each proband’s first-degree relatives were evaluated (Hinton et al., 2007). When additional affected family members were identified, sampling was extended to include their first-degree relatives. Written informed consent was obtained from each participant or participant’s parent or guardian. Assent was obtained from pediatric participants when appropriate. This study was approved by the Institutional Review Board at Cincinnati Children’s Hospital Medical Center.

**Table 1:** Description of participants.

	Family 5	Family 9	Family 14	Family 22	All
*n*	18	16	10	8	52
Sex (%M)	56%	63%	50%	38%	53.8%
Race (% White)	100%	75%	100%	100%	92.3%
% CVM	22%	31%	50%	38%	32.7%

### Echocardiographic Analysis

Cardiac phenotype was determined by cross-sectional 2-dimensional and Doppler transthoracic echocardiography on all participants using Hewlett-Packard Sonos 5500, General Electric Vivid 5 or Vivid 7 systems as previously described (Hinton et al., 2007, 2009). A detailed echocardiographic protocol previously described was used to assess cardiovascular structures (Cripe et al., 2004). A single experienced echocardiographer interpreted echocardiograms (Cripe et al., 2004; Hinton et al., 2007).

### Whole Exome Sequencing (WES)

Whole exome sequencing was performed at the Genetic Variation and Gene Discovery Core of Cincinnati Children’s Hospital. One ug of dsDNA (blood) was used. Quantity was determined by Invitrogen Qubit (Life Technologies, Grand Island, NY, United States) high sensitivity spectro-fluorometric measurement. DNA was sheared by sonication on a Diagenode Bioruptor (Diagenode Inc., USA North America, Denville, NJ, United States). Library construction was performed using Illumina TruSeq DNA Sample Preparation kit (Illumina Inc, San Diego CA, United States) with a size selection at 350 bp post adapter ligation. One ug of genomic library was recovered for exome enrichment using Nimblegen SeqCap EZ Human Exome v2 kit (Roche Nimblegen, Inc, Madison, WI, United States). Enriched libraries were sequenced on an Illumina HiSeq2000 (Illumina, Inc., San Diego, CA, United States), generating at least 30 million paired end reads of 125 bases each per sample, corresponding to an average coverage of 60X.

#### Variant Calling

Variant calling was performed with Genome Analysis Toolkit v3.5-0 (McKenna et al., 2010). Samples were individually pre-processed by realigning reads around putative indels using IndelRealigner tool, marking putative PCR duplicate reads with Picard’s MarkDuplicates tool and by recalibrating base quality scores with BaseRecalibrator tool. Following pre-processing, samples were called with HaplotypeCaller and the resulting gVCFs were jointly processed with GenotypeGVCFs to generate initial variant calls such that regions which differed from the reference genome (alternative allele) for at least one sample in the batch had variants called in all individuals. Variants were then filtered using Variant Quality Score Recalibration (McKenna et al., 2010).

### Data Analysis

#### Quality Control

To reduce the chance of false positives, quality control was employed (Martin et al., 2014). Briefly, at an individual call level, each call had to have coverage ≥20X but ≤250X and a quality score of ≥20. Variant calls which did not meet this threshold were blanked. Indels and variants which had call rates less than 80% were removed. To minimize sequencing artifacts, we eliminated variants for which the alternative allele was called in more than 30% of the individuals. Only autosomal variants were considered because x-linked inheritance was not indicated. CNVs were also not assessed.

#### Bioinformatic Prediction of Deleteriousness

To quantify the bioinformatic evidence of functional impact, we used Combined Annotation Dependent Depletion (CADD) scores (v1.3) (Kircher et al., 2014). This tool was chosen because it allowed evaluation of both missense and loss of function (stop gain/loss, splicing) as well as regulatory regions. Phred scores ≥20 were considered as evidence of a deleterious effect.

#### Prioritization of Variants

##### Conventional approach

We sought to identify variants which were rare, i.e., minor allele frequency (MAF) ≤1% in all reported 1000 Genomes (Genomes Project Consortium et al., 2015) and ExAC populations (Lek et al., 2016). Such variants would be consistent with the reported prevalence of CVM (2–5%) (Benson, 2002). We identified those that had informatics support of a deleterious effect by selecting variants with Phred scores ≥20. Variants present in the heterozygous or homozygous state in the proband meeting these criteria were considered as rare variants with informatics support (RVIS). The list of RVIS was further analyzed for the degree of sharing across probands at the variant and gene level, loss of function variants (predicted stop gain), and ClinVar pathogenic variants to further reduce the number of variants under consideration. In addition because HLHS had by hypothesized to occur when 2 copies of a CVM variant are carried, homozygous variants were also reviewed.

##### Co-segregation approach

To identify co-segregating variants (C-SV) in each family, we performed 2 point linkage analyses of variants, with a dominant model and 80% penetrance and a 1% disease mutant gene frequency using SUPERLINK (Silberstein et al., 2006). We selected 2 point rather than multipoint linkage because 2 point linkage permitted evaluation of all variants present in probands, whereas multipoint linkage would have required removing variants in linkage disequilibrium (Evans and Cardon, 2004). The selection of a dominant model was based on the fact that each pedigree had multiple individuals. Further, prior studies have suggested that BAV, a common phenotype in our families is dominantly inherited (Huntington et al., 1997; Wessels et al., 2005). The penetrance was selected to be 80% because prior studies suggested reduced penetrance (Huntington et al., 1997) and there was incomplete familial transmission of CVM in these families. We also evaluated a model using 50% penetrance. In these models, the LOD scores were lower but the most strongly C-SV was consistent with the model using 80% penetrance (data not shown). We opted to use the same model for all families so that we could sum the LOD across families to obtain a cumulative LOD score. Individuals with CVM were considered affected; individuals without CVM were considered unaffected. Individuals with unknown phenotype (labeled with ? in the pedigrees) were scored as missing. LOD were summed across the families to create a cumulative LOD score. However, similar inference was made when evaluating family specific LODs. Assuming two fully informative markers, the theoretic maximum LOD per family was 2.7, 2.1, 1.2, and 0.9 for Family 5, 9, 14, and 22, respectively. However, as our prior data suggests as single variant is likely not sufficient (e.g., reduced penetrance), the bilineal nature of some of the pedigrees, and the information content of SNPs we would not expect to obtain such high LODs.

## Results

### Cardiovascular Phenotypes of Participants

**FIGURE 1:**
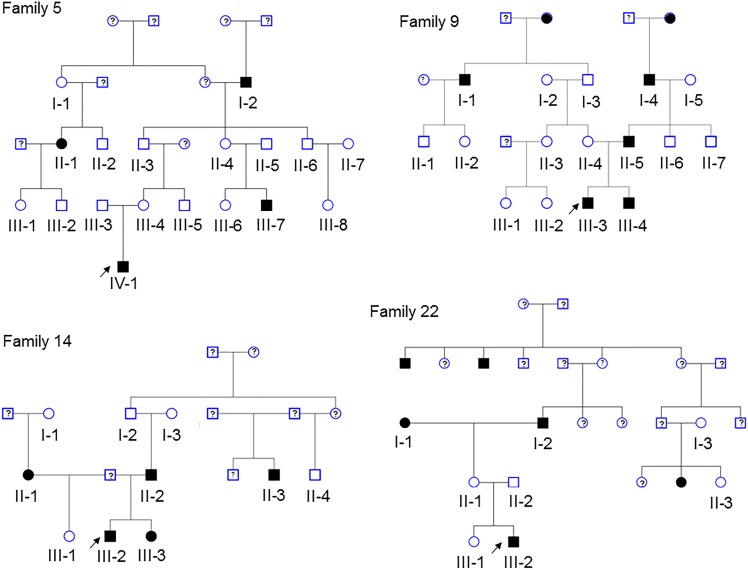
Pedigrees of the 4 families in the study. Arrow denotes probands, each of which has HLHS. Solid shapes denote an individual with CVM. Open symbols denote unaffected individuals with normal echocardiogram and a DNA sample. Individuals with a ? have unknown phenotype status and were not genotyped. Exome sequencing was performed in 52 participants.

Pedigrees of 4 multiplex families are illustrated in Figure 1. CVM phenotype was present in 17 of 52 (33%) (Supplementary Table 1). HLHS was present in 5 participants. Varied CVM phenotypes were observed in other family members, either alone or in combination. BAV was present in 4 family members, but 7 additional family members had abnormal aortic valve that did not meet criteria for BAV. Abnormalities of the aorta were present in 4 family members including coarctation of the aorta in 2 participants and dilated aorta in 2 participants. Ventricular septal defect and atrial septal defect in combination with abnormality of the mitral valve were each present in a single individual. These findings underscore the importance of screening relatives of HLHS patients to identify those at risk for latent disease.

### Rare Variants With Informatics Support (RVIS) Are Common in HLHS Probands

**FIGURE 2:**
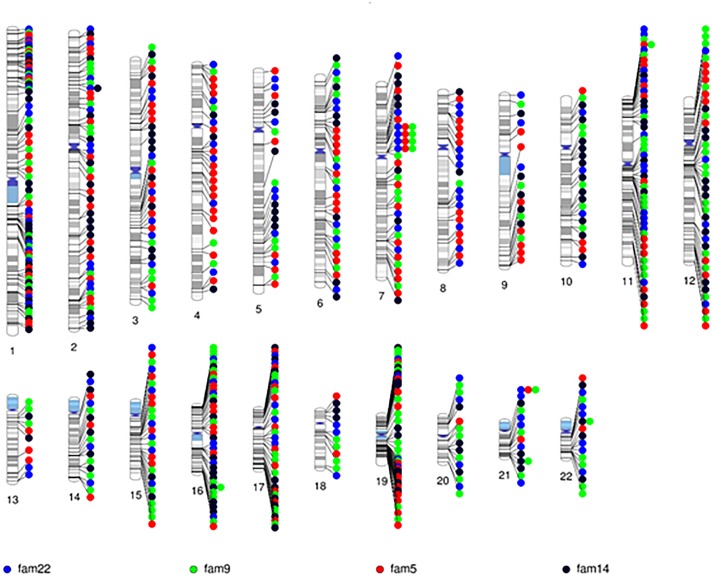
Chromosomal distribution of the rare variants with informatics support (RVIS) (MAF ≤ 1% and CADD ≥ 20) identified across the genome in 4 probands. Findings reveal an abundance of variants (*n* = 702) with informatic support. Stacked dots represent sharing between probands.

After quality control, there were 108,048 variants called in the 52 individuals. Among these variants, 31,867 were heterozygous or homozygous for the alternative allele in at least 1 of the 4 probands with no additional filtering. Following the conventional approach, 702 RVIS (668 genes; 159–194 variants per proband) were identified (Figure 2 and Supplementary Table 2). Limiting the results to variants seen in the homozygous state, a single variant (rs139011641, *MUC6*) was identified in a proband. Of the 702 RVIS, only 9 were seen in at least 2 of the probands and none in all 4 (see Supplementary Table 3). Further, there were 27 genes which harbor RVIS in more than one family (2 families *n* = 24, 3 families *n* = 3; Supplementary Table 4). Importantly, many of these genes do not harbor the same variant across families. Within this list of 702 variants, there are 28 stop gain variants (Supplementary Table 5) and 5 variants reported in ClinVar as pathogenic or likely pathogenic (Supplementary Table 6). Limiting the results to variants with MAF ≤ 0.1% reduced the number of variants by ∼50% (*n* = 358) while limiting the results to MAF ≤ 0.01% resulted in 296 variants. However, as a practical matter, this variant list is still prohibitively long for biological evaluation.

### RVIS Rarely Co-segregate With CVM in Families

**FIGURE 3:**
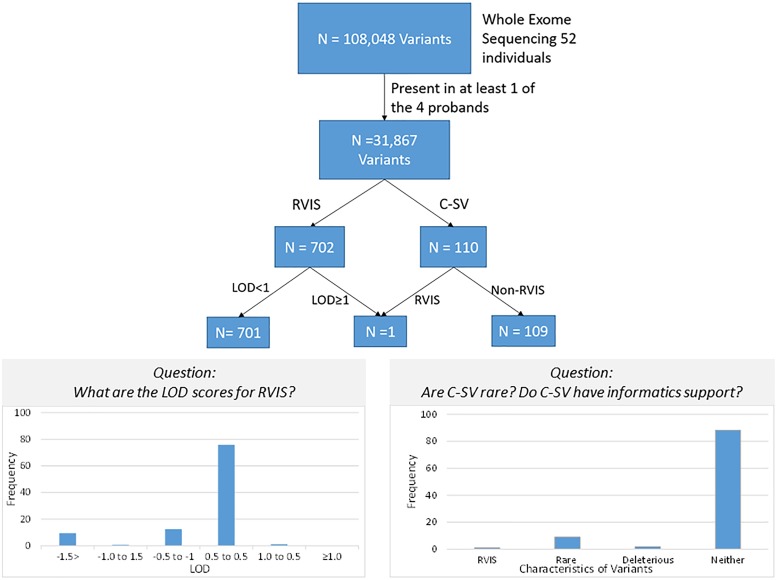
Schematic of variant discovery. We used two approaches for variant discovery. First, a conventional approach identified rare variants with informatic support (RVIS: Minor allele frequency ≤ 0.01 and CADD prediction ≥ 20 for deleterious effect). Second, a co-segregation approach identified co-segregating variants (C-VS, LOD ≥ 1.0). Only one variant overlapped both approaches. Overall for RVIS, there was little evidence of co-segregation as less than 2% of RVIS had a LOD ≥ 0.5. For the C-VS, only 10% were rare and less than 3% were predicted to be deleterious.

All 702 RVIS were also present in at least one parent (suggesting these were not new mutations). We determined the cumulative LOD scores of the RVIS variants across the 4 families. The maximum LOD was 1.3 for a variant on chromosome 4, rs200183228, a non-synonymous variant in *SORCS2*. No other variants had cumulative LOD ≥ 1.0 (nor family specific, data not shown) and only 8 other variants had LOD > 0.5 (Supplementary Table 7 and Figure 3). However, nearly 25% of variants had LOD < -0.5 (Figure 3). Notably, 11 of these variants had LOD < -2, which would be sufficient to exclude a locus under the specified genetic model.

### Characteristics of Variants That Co-segregate With CVM in Families (C-SV)

**FIGURE 4:**
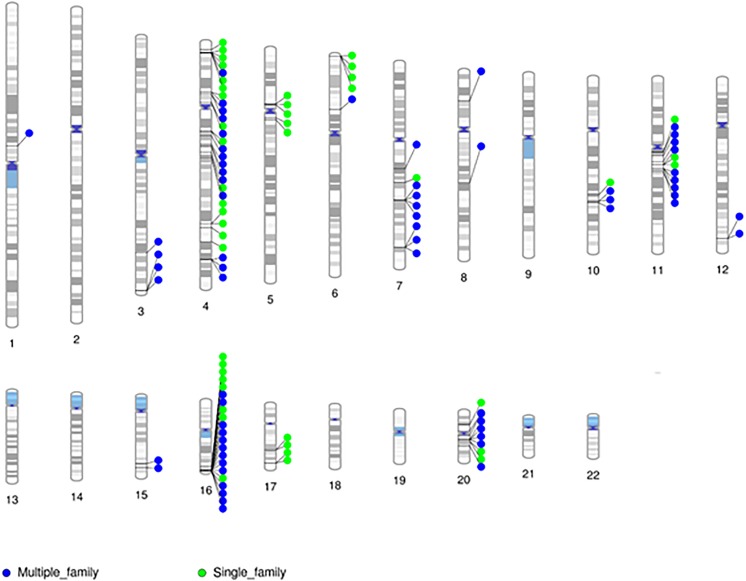
Chromosomal distribution of segregating markers (cumulative LOD ≥ 1.0; *n* = 110) demonstrates the presence of multiple regions contributing to CVM.

Given the minimal evidence of linkage among RVIS, we then evaluated linkage across 31,867 variants seen in at least one proband (Supplementary Figure 1). The larger families (Family 5 and 9) exhibited multiple variants with moderate evidence of linkage (LOD ≥ 1.0) while the smaller families (Family 14 and 22) did not (Supplementary Table 8). The highest cumulative LOD score was 2.0 for rs34053053 (chromosome 11, an intronic variant in *CTTN*). There were 9 additional C-SV with cumulative LOD ≥ 1.5. Interestingly, 2 of these variants were in also in *CTTN* (rs2298396 and rs2298397, both LOD = 1.7) and 3 variants were in *GSTP1* (rs1695, rs1871042, and rs4891, all LOD = 1.6). One variant was present in *ARAP1-AS2* (rs12575364), *PCLO* (rs12668093), *SORCS2* (rs28531835), and *MS4A5* (rs708229). The phenotypes of the affected individuals carrying these variants are varied with no clear pattern of specific variants being associated with abnormalities in the aortic valve, aorta, mitral valve or other CVM (Supplementary Table 9). There were 110 C-SV with cumulative LOD ≥ 1.0 (Figure 4). These variants were distributed across 14 chromosomes, with 5 chromosomes (4, 16, 11, 20, and 7) having 9 or more C-SV.

Based on the minimal overlap between RVIS and C-SV, we then sought to characterize population frequency and informatics support as separate components (Figure 3). Strikingly, 97% of C-SV are common (MAF > 1%) with the rarest variant occurring at a frequency = 0.003. Moreover, 90% of C-SV did not have informatics support of functionality. Among the 10 variants with LOD ≥ 1.5, all variants are common and none are predicted to be functional (Supplementary Table 8).

## Discussion

In this study, we used multiplex families to compare 2 methods of variant identification for exome data. A conventional approach that relies on documentation of RVIS was compared to an approach based on variant co-segregation (C-SV) with CVM in families using existing linkage analysis methods. In terms of variants identified by the 2 approaches, we found little overlap. Specifically, we found that while emphasis on RVIS identified a large number of variants in probands, few of these variants co-segregated with disease in our families. Surprisingly, among C-SV, the great majority were non-RVIS. Lastly, we found that C-SV are usually common with multiple variants per family. Taken together, these results support the value of family based studies for CVM discovery as these studies can evaluate both C-SV as well as RVIS. Our findings support the use of linkage analyses in multiplex families for left sided CVM gene discovery with exome sequencing data to evaluate more complex inheritance models.

When following the conventional approach for exome data, we identified a large number of RVIS in probands. This is not surprising since pathogenic variants in actionable genes commonly occur in unaffected individuals (Dorschner et al., 2013; Tabor et al., 2014; Lindor et al., 2017). In addition, finding of a large number of RVIS is consistent with prior case series and trio studies for left sided CVM (Zaidi et al., 2013; Homsy et al., 2015; Priest et al., 2016; Jin et al., 2017). Further complicating the discovery efforts for case series is the marked genetic heterogeneity for CVM as demonstrated from linkage (Benson et al., 1998; Martin et al., 2007; Hinton et al., 2009; McBride et al., 2011), association (Gago-Diaz et al., 2017), and exome studies (Zaidi et al., 2013; Homsy et al., 2015; Jin et al., 2017; Li et al., 2017). To overcome the challenges of heterogeneity, researchers have focused on enrichment, which is often restricted to *de novo* variants (Homsy et al., 2015), protein truncating variants (Sifrim et al., 2016), or gene lists (Kathiresan et al., 2009; LaHaye et al., 2016; Blue et al., 2017; Szot et al., 2018). Even with these strategies, a large proportion of CVM has unknown etiology (Benson et al., 2016; Jin et al., 2017; Wilsdon et al., 2017; Russell et al., 2018; Szot et al., 2018), suggesting alternative strategies are needed.

Importantly, we found that most (>99%) C-SV were not RVIS and thus would have been missed with conventional exome workflows. Other studies that have included family data, have noted that many RVIS do not co-segregate (Arrington et al., 2012; Preuss et al., 2016; Blue et al., 2017). Unfortunately, non-RVIS were not considered in these prior studies. Further, the failure of informatics to support a role for 90% of the C-SV is not surprising as studies have raised concerns about the accuracy of these tools for protein coding changes (Miosge et al., 2015; Mahmood et al., 2017). The situation for informatic prediction becomes more challenging for non-protein coding variation which is based cellular transcriptomes (Sloan et al., 2016). Indeed, the utility of cell based transcriptomics to understand the dynamics underpinning organogenesis where multiple cell types must interact with each other is unclear. Given the lack of RVIS that segregate, these results challenge the assumption that informatics will be the best initial filter for CVM gene discovery.

Following ACMG guidelines, we sought to determine if variants and the genes identified through linkage analysis were biologically plausible. We found 3 chromosomal regions (chromosome 4, 7, and 11 encompassing 6 genes) with LOD ≥ 1.5, and each of these regions had support in 2 families. The chromosome 11 region spanned 60198328 to 72409189 base pairs with variants attributed to 4 genes: cortactin (*CTTN*, 3 variants), Glutathione S-Transferase Pi 1 (*GSTP1*, 3 variants), Membrane Spanning 4-Domains A5 (*MS4A5*, 1 variant), and ARAP1 Antisense RNA 2 (*ARAP1-AS2*, 1 variant). The variant with the strongest evidence of co-segregation, rs34053053, is located in an intron of CTTN but alters expression of PTPRF Interacting Protein Alpha 1 (*PPFIA1*) (GTEx Consortium, 2015). *PPFIA1* is part of a family of scaffolding proteins involved in focal adhesion turnover, cell migration, and tissue organization (Asperti et al., 2009; Astro et al., 2014, 2016). *PPFIA1* regulates integrin (Asperti et al., 2010), is critical for heart development (Parker and Ingber, 2007), and affects vascular morphogenesis in developing zebrafish (Mana et al., 2016). Another gene on chromosome 11 is *GSTP1*, an oxidative stress-related detoxification enzyme. The 3 segregating variants were predicted to be eQTLs for *GSTP1* using GTex (GTEx Consortium, 2015). Prior work has identified *GSTP1* variants associated with CVM (Nembhard et al., 2017). *MS4A5* is expressed primarily in testes but is detectable in heart and is speculated to be involved in signal transduction. Also included on chromosome 11 is *ARAP1-AS2*, a non-coding RNA. Chromosome 4 contains Sortilin Related VPS10 Domain Containing Receptor 2 *(SORCS2).* Notably, *SORCS2* was identified both by segregation variants and RVIS, but the strongest segregating variant was non-RVIS. In mice, *Sorcs2* is expressed in mesodermally derived structures of the heart prior to E15.5 (Glerup et al., 2014; Boggild et al., 2016) as well as after myocardial ischemia (Siao et al., 2012). Chromosome 7 contains Piccolo Presynaptic Cytomatrix Protein (*PCLO)*. *PCLO* is down regulated in the hearts of mice lacking cardiac myosin binding protein C (Eijssen et al., 2008). Thus, a limited number of biologically plausible candidate genes, which deserve further consideration, were identified using linkage analyses.

Beyond the identification of novel candidates, utilization of co-segregation provides evidence that multiple common variants contribute to left sided CVM, aka complex inheritance. The finding that multiple rather than a single variant may be required is consistent with work in humans (McBride et al., 2005; Martin et al., 2007; Hinton et al., 2009; Li et al., 2017) and mice (Liu et al., 2017). Prior studies have reported associations between common variants and CVM (Stevens et al., 2010; Qian et al., 2014; Hanchard et al., 2016); however, these studies did not evaluate the functional nature of these variants. Thus, it is not clear whether the common variants are simply in linkage disequilibrium with nearby rare variants. The role of common variation in CVM seems to be in direct contrast to the recommendations of ACMG which suggest that a variant should occur at a frequency lower than the disease prevalence (Richards et al., 2015). While this recommendation is appropriate for Mendelian inheritance where a single variant is sufficient, when multiple variants are required for disease this may not be the case. For example, considering the dominant example where a single variant is sufficient in the heterozygous state, a variant with an allele frequency of 1% would be expected to be seen in 2% of the population, similar to the population estimates of CVM. However, 2 independent variants each with an allele frequency of 5%, would be expected to co-occur infrequently, i.e., in ∼1% of the population. Further, combinations of common and not so common variants may also result in co-occurrence rates consistent with disease prevalence. The challenge with such a scenario is that the number of possible variants that could contribute to disease could increase exponentially. Moreover, reduced penetrance may result in increased frequency in the general population. Thus, for traits which exhibit complex inheritance, utilization of multiplex families may be essential for disentangling the genetic etiology.

There are several limitations to our study. First, while our data clearly supports a disconnect between C-SV and RVIS, we did not demonstrate that non-RVIS were causal. However, if CVM is a complex trait influenced by multiple variants, then evaluation of causality becomes more challenging, as all causal variants must be jointly evaluated. Second, we do not know the extent to which our results, obtained from families ascertained by probands with left sided heart malformations, are generalizable to other CVM phenotypes. Third, our study was a relatively small sample, with 52 individuals from 4 families. Further, three of the four families have CVM in two distinct lines of descent. While this makes variant discovery challenging, given the not so rare nature of CVM (Benson, 2002; McBride et al., 2005) such occurrences are not unexpected but would be missed without detailed phenotyping in extended families. Clearly, additional studies using multiplex CVM families are warranted. Lastly, we did not evaluate copy number variation (CNV) which have been recognized to contribute to CVM (Costain et al., 2016; Hussein et al., 2018).

Taken together these results suggest that the approach to whole exome/genome variant discovery for left sided CVM needs to be reconsidered. Specifically, the results presented here as well as findings of prior studies (McBride et al., 2005; Martin et al., 2007; Hinton et al., 2009; Liu et al., 2017) suggest that multiple genetic variants contribute to disease development. Given such a scenario, the challenge will be how to narrow the list of possible variants. We found that use of linkage narrowed the variant list to a manageable level with 10 variants exhibiting LOD ≥ 1.5. Results from our linkage analyses suggest that there may be multiple variants within a linkage region as well as multiple independent loci contributing to disease. These combinatorial effects may be due to alterations in chromatin accessibility or looping (Tolhuis et al., 2002; Spilianakis and Flavell, 2004; Vernimmen et al., 2007; Jing et al., 2008; Smemo et al., 2014), long range gene regulation (which can occur on the same or different chromosomes) (Spilianakis et al., 2005; Lomvardas et al., 2006) or genes which individually contribute to disease etiology (Winston et al., 2012). The next logical step would be to evaluate how combinations of variants contribute within these families and if these combinations help explain the phenotypic heterogeneity present. Unfortunately, the sample size of the current study does not provide sufficient power to evaluate combinatorial effects. Once hypotheses have been generated, then generalizability of variants identified in families can be evaluated in the case series and trios for which exome or genome data are available such as the Pediatric Cardiac Genomics Consortium (Pediatric Cardiac Genomics Consortium et al., 2013). Additionally, when considering how to biologically validate variants, the multigenic context must be considered. Fortunately, with the advances in CRISPR/cas9 genome editing, multigenic mouse models can be evaluated (Liu et al., 2017).

In summary, these results suggest that for left sided CVM, conventional identification of candidate variants using allele frequency and predicted informatic functionality may miss a large proportion of variants co-segregating with CVM. This is likely due to the complex genetic basis of CVM for many families. For birth defects such as CVM that exhibit strong familial transmission, yet for which single variants are not sufficient, utilization of linkage analyses could be a powerful tool to identify novel candidates missed by conventional strategies.

## Web Resources

• CADD: http://cadd.gs.washington.edu/
• Chromosome plots: http://visualization.ritchielab.org/pheno grams/plot

## Author Contributions

LJM developed the question, performed and oversaw analyses, interpreted the results, and drafted the manuscript. DWB developed the question, critically revised the manuscript. VP performed analyses and aided in interpretation.

## Conflict of Interest Statement

The authors declare that the research was conducted in the absence of any commercial or financial relationships that could be construed as a potential conflict of interest.

## Supplementary Material

FIGURE S1 Distribution of cumulative LOD in RVIS and non-RVIS variants. Distributions are presented as Violin plots.

TABLE S1 Phenotypic characteristics of affected individuals.

TABLE S2 Rare variants with informatic support.

TABLE S3 RVIS which are shared across families.

TABLE S4 Genes harboring RVIS present in more than one family.

TABLE S5 Loss of function RVIS.

TABLE S6 RVIS reported to be pathogenic in ClinVar.

TABLE S7 RVIS with cumulative LOD ≥ 0.5.

TABLE S8 Variants which exhibit evidence of segregation with CVM (LOD ≥ 1.0).

TABLE S9 Variants which exhibit evidence of segregation with CVM (LOD ≥ 1.5) and phenotypes associated.
